# Activity Time Budgets—A Potential Tool to Monitor Equine Welfare?

**DOI:** 10.3390/ani11030850

**Published:** 2021-03-17

**Authors:** Ulrike Auer, Zsofia Kelemen, Veronika Engl, Florien Jenner

**Affiliations:** 1 Anaesthesiology and Perioperative Intensive Care Medicine Unit, Department of Companion Animals and Horses, University of Veterinary Medicine Vienna, Veterinaerplatz 1, 1210 Vienna, Austria; veronika.engl@hvu.vetmeduni.ac.at; 2 Equine Surgery Unit, University Equine Hospital, Department of Companion Animals and Horses, University of Veterinary Medicine Vienna, Veterinaerplatz 1, 1210 Vienna, Austria; Zsofia.Kelemen@vetmeduni.ac.at

**Keywords:** horse, equine, activity, time budget, behaviour

## Abstract

**Simple Summary:**

Horses’ behavior is a good indicator of their welfare status. However, its complexity requires objective, quantifiable, and unambiguous evidence-based assessment criteria. As healthy, stress-free horses exhibit a highly repetitive daily routine, horses’ time budget (amount of time in a 24 h period spent on specific activities) can assist in equine welfare assessment. A systematic review of the literature yielded 12 papers that assessed equine time budgets for eating, resting and movement for a minimum of 24 continuous hours. A total of 144 horses (1–27 years old), 59 semi-feral and 85 domesticated horses, are included in this review. The reported 24 h time budgets for eating ranged from 10% to 66.6%, for resting from 8.1% to 66%, for lying from 2.7% to 27.3%, and for movement from 0.015% to 19.1%. The large variance in time budgets between studies can largely be attributed to differences in age and environmental conditions. Management interventions (free access to food, increased space, decreased population density) in domesticated horses yielded time budgets similar to semi-feral horses. The data support the importance of environmental conditions for horses’ well-being and the ability of time budgets to assist in monitoring horses’ welfare.

**Abstract:**

Horses’ behavior can provide valuable insight into their subjective state and is thus a good indicator of welfare. However, its complexity requires objective, quantifiable, and unambiguous evidence-based assessment criteria. As healthy, stress-free horses exhibit a highly repetitive daily routine, temporal quantification of their behavioral activities (time budget analysis) can assist in equine welfare assessment. Therefore, the present systematic review aimed to provide an up-to-date analysis of equine time budget studies. A review of the literature yielded 12 papers that fulfilled the inclusion criteria: assessment of equine time budgets for eating, resting and movement for a minimum of 24 continuous hours. A total of 144 horses (1–27 years old), 59 semi-feral and 85 domesticated horses, are included in this review. The 24 h time budgets for foraging or eating (10–6.6%), resting (8.1–66%), lying (2.7–27.3%), and locomotion (0.015–19.1%) showed large variance between studies, which can largely be attributed to differences in age and environmental conditions. Management interventions in domesticated horses (ad libitum access to food, increased space, decreased population density) resulted in time budgets similar to their (semi-)feral conspecifics, emphasizing the importance of environmental conditions and the ability of time budgets to assist in monitoring horses’ welfare.

## 1. Introduction

Animal welfare is a multifaceted, continuously evolving concept at the interface of science and society, influenced by ethical, economic, cultural, and political concerns [1,2,3,4,5,6]. Its multidimensionality, both from a scientific and societal perspective, requires objective, quantifiable, and unambiguous evidence-based parameters to assess animal welfare and inform guidelines and policies [4,5,6,7,8,9,10].

As welfare is a subjective individual experience, the animal welfare scientific community has moved progressively from input or resource-based measures, such as housing type or the amount of food provided to the animal, toward more output or animal-based assessments that not only evaluate the quality of the environment in which an animal is kept but also its physical and psychological condition and its ability to express the full repertoire of species-appropriate behaviors [6,9,11]. Therefore, behavior is increasingly used as an indicator of welfare because an animal’s behavior can provide valuable insight into its subjective state [6,12,13]. The behavior of horses is defined by their niche as a prey species and highly social grassland dwellers with strong group fidelity who, in their natural habitat, are continual grazers with ultradian activity patterns roaming areas of land up to 78 km^2^ [14,15,16,17]. Healthy, stress-free horses divide their time between activities that allow them to satisfy their basic requirements of food, movement, and rest, and exhibit a highly repetitive, individual, daily routine with almost identical time patterns of behavior from day to day [15,18,19]. Accordingly, the amount of time an animal engages in behavioral activities (time budget) is considered a very informative welfare indicator [6,9,15,20,21,22,23,24]. To this end, domesticated horses are often compared to feral or wild conspecifics. Thereby, feral horses can be used as a benchmark for comparison, not as gold standard for optimum welfare [6,9,15,20,21,22,23,24]. Indeed, domestic horses, given the opportunity to display species-appropriate behavior and an environment sufficiently reflecting a natural habitat, display time budgets similar to those of wild horses [4,15,23,25]. However, domestic horses are kept in a variety of housing systems that offer more or less adequate environmental conditions with differing levels of physical freedom, often with regimental feeding and limited foraging and social opportunities [14,15]. Thus, differences in the time budgets of domesticated horses compared to feral or wild conspecifics are currently used as a measure of compromised welfare [6,15,23,24,26,27].

As equine behavioral activity displays 24 h and circadian variation, measuring time budgets requires detailed surveillance over several days [9,17,28,29]. Traditionally, this has been done by direct observation which was prone to observer bias, often limited to daylight hours and too time and resource-intensive to be feasible for welfare assessment. However, recent advances in biotelemetry and artificial intelligence (AI) based on sensor (mostly accelerometer) or video analysis provide increased objectivity and enable remote recording of behavioral data, longer observation periods, and quantification of behaviors with higher accuracy and temporal resolution than the human eye allows [30,31,32]. Hence, time budget analysis, as an objective, quantitative measure of behavior, now has the potential to become a useful, reliable tool for on-farm assessment of equine welfare and comparison of welfare under different environmental conditions. Therefore, the present review aimed to provide an up-to-date analysis of equine time budget studies and synthesize evidence relating to the effect of housing and management systems on horses’ behavioral time budgets.

## 2. Materials and Methods

This review was carried out in accordance with the Preferred Reporting Items for Systematic Reviews and Meta-Analyses (PRISMA) guidelines [33].

### 2.1. Data Sources and Searches

**Figure 1:**
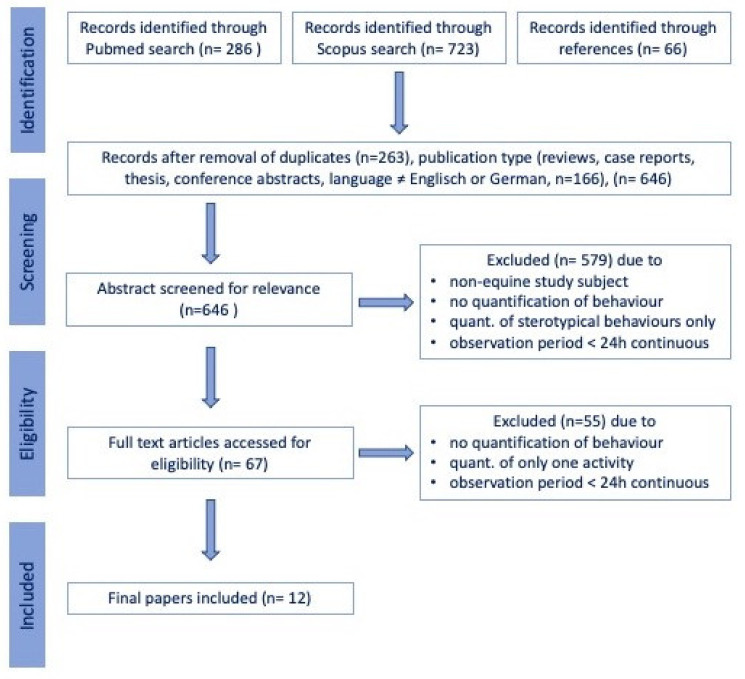
Flow chart illustrating the selection of studies included in the review.

Scientific peer-reviewed articles were identified through a systematic search in the PubMed (National Institutes of Health. PubMed (Database). Bethesda, MD, USA: National Library of Medicine; https://pubmed.ncbi.nlm.nih.gov, accessed on 25 February 2021) and Scopus (Elsevier, Amsterdam, The Netherlands; https://www.scopus.com, accessed on 25 February 2021) electronic databases searching for the terms “((“time budget” OR “activity budget” OR “activity tracking”) OR ((sleep OR sleeping OR resting OR lying OR eating OR foraging) AND (behaviour OR behavior OR time OR video OR sensor OR gyroscope OR accelerometer))) AND (horse OR equine OR pony OR horses OR ponies OR “equus caballus”)“ in title or abstract, with no restriction on publication date, in November 2020. The study selection process was carried out by the first and last author following the procedure detailed in Figure 1, excluding papers that upon closer inspection, did not study the time horses spend on specific activities or did not include a minimum of 24 h uninterrupted observations. Any disagreement between the authors on the studies to be included in the review was resolved during a consensus meeting.

All quantitative or qualitative randomized controlled trials, observational studies, and case series focused on equine time budgets or activity quantification written in English or German were included. The following exclusion criteria were set: (a) non-peer-reviewed publication, (b) conference/seminar abstract only published, (c) dissertation, thesis, review, commentary, or single case report, (d) the article was not written in English or German.

### 2.2. Data Extraction and Risk of Bias Assessment

Information on the population, intervention, comparison, outcome, and study design (PICOS) was retrieved from the articles, and the risk of bias of selected studies was assessed using a modification of the Evidence Project risk-of-bias tool [34,35,36].

## 3. Results

A total of 286 articles were identified in PubMed, 723 additional papers in Scopus, and another 66 based on references, yielding a total of 1075 articles. After removing duplicates, reviews, commentaries, single case reports, books and non-English or German articles, 646 papers remained. Following the exclusion of papers that did not focus on equine activity tracking or time budgets, but on different species or other behavioral observations or used an observation period of less than 24 h per day, 12 articles remained included in the qualitative synthesis [18,28,37,38,39,40,41,42,43,44,45,46]. A total of 6 (50% of the total) articles were classified as observational studies [18,28,37,38,39,45], 5 articles (41.6% of the total) as prospective, non-blinded, non-randomized case series [41,42,43,44,46], 1 article (8%) as prospective, non-blinded, non-randomized controlled trial [40] and none as randomized controlled trials (Table 1).

Risk-of-bias assessment (Table 1) revealed the lack of a control or comparison group (only 8.3% of the articles fulfilled this criterion), random assignment of participants to intervention (8.3% of the articles fulfilled this criterion), random selection of participants for assessment (none of the articles fulfilled this criterion), as the most critical concerns. Further limitations of the papers included in this review are the small sample sizes (4–22 horses) and the variable observation methods which were restricted to manual observation in the field in 33% [18,37,38,39] and manual behavior scoring from video in 50% [40,41,42,43,45,46]. Only 25% used biotelemetry devices (25%) [28,44,45]. Manual quantification of observed behavior was carried out in 83.3% (10/12 studies) of the studies using scan sampling in 33% (4/12 studies) [18,37,38,46], focal sampling [39,43], and instantaneous sampling in 16.6% (2/12 studies) [40,41] each, and ad libitum sampling [45] and unspecified methodology [42] in one paper. In addition, the ethogram, based on which behaviors were categorized, was not provided in 25% [39,41,42], and the video analysis software was not detailed in 41.6 % [40,42,44,45,46] of the articles. Data analysis was limited to descriptive statistics in the observational studies [18,28,37,38,39,45] and the other 50% of the included papers detailed further statistics [40,41,42,43,44,46]. The large variance in study methodology and data presentation did not allow combining the data for meta-analysis.

A total of 144 horses are included in the present systematic review comprising ages between 1 and 27 years (Table 2) [18,28,37,38,39,40,41,42,43,44,45,46]. Three papers (25% of the total) studied free-ranging (300–335 ha area) (semi-)feral horses [18,37,38], two (17% of the total) Przewalski horses that were bred in zoos and living in a 44 ha semi-reserve [28,39], and seven (58% of the total) domesticated horses [40,41,42,43,44,45,46]. All studies determined the 24 h time budgets for foraging or eating (10–66.6%), resting (8.1–66%) and locomotion (0.015–19.1%), but only 9 (75% of the total) specifically quantified lying (2.7–27.3%) (Table 3) [18,37,38,39,40,42,43,45,46]. Two of the three studies on free-ranging horses [18,37] were focused on the effect of age and sex on time budgets and one on environmental and seasonal influences [38].

The time budget of free-ranging horses was divided between 13–66.6% eating or foraging (weaned, grazing horses: 50.82–66.6%), 8.1–29.3% resting, 2.7–15.5% lying, and 4.3–13.4% locomotion [18,37,38]. Age had a significant effect on the time budgets of free-ranging horses, with foals until weaning spending significantly more time sleeping while lying flat, while expectedly, after weaning, the time spent foraging increased [18]. Stallions spent more time standing alert and moving rapidly but less time foraging than mares [37].

Przewalski horses kept under more confined conditions (44 ha semi-reserve) dedicated 29.8–46.4% of their time budget to eating or foraging, 36.4–36.6% to resting, 5.3% to lying, 7.4% to locomotion, and 10.2% to other behaviors such as drinking, self-grooming, and playing (Table 3) [28,39]. Behavioral analysis by automated tracking revealed a complex diurnal and ultradian rhythmicity and seasonal variations of activity patterns [28].

Four of the seven studies on domesticated horses [41,42,43,45] studied the effect of different feeding regimes on equine time budgets, and one study evaluated the effect of postoperative pain [40], stocking density [46] and turn-out management together with paddock size [44]. The time budget of domesticated horses was divided between 10–64% eating or foraging, 15.6–68% resting, 3–27.3% lying, 0.015–19.3% (in horses not confined to a stable: 2.5–19.3%) locomotion and 2–11.5% other behaviors such as drinking and self-grooming (Table 3) [40,41,42,43,44,45,46]. The large variance in activity time budgets can in part be explained by the age range of the horses included in the various studies, as young horses are resting more in a recumbent position than adults [43,46].

The feeding regime significantly affected the time budget of horses [41,45]. Regular feeding times and coordinated rations led to a significant reduction in the time spent on feeding. Offering the hay ration in hay bags increased the time spent feeding but to a lesser extent than ad libitum feeding [41,45]. The diurnal and ultradian rhythmicity of feeding of wild and feral horses could also be observed in domestic horses with more time spent feeding during daytime [42,44]. Paddock size and stocking density had no influence on feeding time but affected time for resting and locomotion with a decreased stocking density showing a positive correlation with locomotion, playing, and self-grooming [46] and a smaller paddock size being associated with decreased locomotion time [44].

## 4. Discussion

Aiming to provide an up-to-date analysis of equine time budget studies, this review included all studies that determined equine time budgets for eating, resting and locomotion over an observation period of at least 24 h [18,28,37,38,39,40,41,42,43,44,45,46]. The reported time budgets of (semi-)feral and domesticated horses show large variances (Table 3). In (semi-)feral horses, the variation in time budgets was primarily attributed to age [18], sex [37] and seasonal influences with correspondingly variable food availability, temperatures, and insect pest densities [28,38]. At this point, it is important to take into consideration that the papers included in this review studied domesticated and semi-feral but not wild horses and hence may not entirely reflect the natural behavior of their wild ancestors.

The differences in the average time budgets between (semi-)feral and domesticated adult horses were especially evident for eating (semi-feral: 50.82–66.6% versus domesticated: 10–64%) and resting (semi-feral: 12.9–29.3% versus domesticated: 15.6–66%, Table 3) [18,28,37,38,39,40,41,42,43,44,45,46]. However, while the limitations of feeding opportunities and feed availability have different causes in wild, (semi-)feral and domesticated horses, offering ad libitum food to domesticated horses increased their time spent eating to levels similar to their (semi-)feral conspecifics (Table 3) [41,42,45], highlighting the importance of management interventions for equine welfare. Furthermore, horses kept in small paddocks or densely populated group pens exhibited significantly increased resting times (Table 3) [44,46]. Decreasing stocking density reduced the high resting times to levels approximating the time budgets seen in other studies (Table 3) and increased locomotion, playing, and self-grooming [46]. This supports the utility of time budgets for monitoring interventions aimed at improving horses’ welfare.

### 4.1. Time Budget Measurement: Methods and Use

Unfortunately, even though Berger et al. already used a telemetric system in 1999 to monitor a group of horses over one year [28], many studies included in this review have not made optimal use of available technology (Table 1). Instead, they used direct observation or video recordings with scan or focal sampling which does not provide a continuous assessment of behavior and thus potentially misses or underrepresents important, infrequent behaviors. Using automated tracking methods instead of visual observation reduces observer bias, improves data resolution, and allows long-term tracking of larger group sizes. This may help to reveal behavioral patterns over more extended periods and determine the influence of environmental conditions on time budgets. Currently, most biotelemetry systems are not yet able to differentiate reliably between different gaits (walk, trot, canter) and between walking and static movements (e.g., stamping, twitching to ward off pests) and thus may determine erroneous gait patterns or too high movement values [32,47]. However, automated tracking methods have been validated in other species (seals, goats, pigs, birds) [30,31,48,49,50] and show great promise for application in equine studies [28,44].

The time budgets of several welfare relevant behaviors, such as foraging, resting, and lying, can already be accurately determined with commercially available sensors and can be used as welfare indicators to identify welfare problems and monitor the success of interventions [32,44]. Furthermore, real-time analysis of equine behavior may also facilitate early detection of health problems, such as colic, lameness or other painful conditions and accelerate therapeutic interventions [4,40,51,52,53,54,55]. Indeed, time budgets for specific behavior have been identified as more sensitive indicators of equine discomfort than repeated direct observation of specific events and postures and thus could facilitate rapid detection of painful conditions and objective, quantitative monitoring of the success of therapeutic interventions [40,54,55].

### 4.2. Time Budget for Feeding and Foraging

Horses’ digestive physiology and anatomy have adapted to their natural diet that is rich in fiber and low in starch and energy [27,54,56]. Free-ranging horses devote the majority of their time to the search for and consumption of food, spending up to 18 h a day foraging, and rarely fast voluntarily for more than 2 to 4 h at a time [27,57]. Thus, the time budget for foraging in grazing (semi-)feral horses ranges from 50.82% to 66.6%, with circadian and seasonal variations depending on climatic conditions and food quality but also differences between daylight and darkness (Table 3) [18,28,37,38,58]. However, many domestic horses have limited access to roughage and are fed restricted amounts of hay and a commercial feed with higher caloric density. Thus, the determined time budget for foraging in the adult domestic horse varies greatly from 16% with rationed feeding [45] to 64% with ad libitum access to hay (Table 3) [41]. One of the papers included in this systematic review also found postoperative pain to reduce feeding time [40] but did not separate the effects of pain from those of general anesthesia. In contrast, another recent study investigating the influence of an iatrogenically-induced acute septic osteoarthritis on behavior found no effect of pain on feeding behavior [50].

Feeding time budgets are relevant for equine welfare because the reduction of the time spent foraging may induce health problems such as gastric inflammation and ulceration. Insufficient eating times have also been associated with the emergence of stereotypes and abnormal behavior, such as increased time spent active walking [6,27,54,57,59,60,61,62]. In contrast, management interventions providing increased foraging opportunities have shown to decrease abnormal behavior [24,59,61,63,64,65,66] and yield time budgets for eating analogous to their wild conspecifics [41,42,45], further confirming that reduced opportunities for foraging may be a source of stress and poor welfare for domestic horses [27,41,45,54,59,60,61,62,66,67,68,69,70,71].

### 4.3. Time Budget for Resting and Sleeping

Adult feral horses spend 12.9–29.3% of their day resting standing and 4.2–15.5% lying in a polyphasic pattern in multiple shorter periods (Table 3) [37,38,72]. However, the time budget for resting, which includes periods of inactivity and sleep, cannot be compared beyond doubt between studies, as there is disagreement whether vigilant standing is part of the resting behavior or activity behavior or forms an independent behavior category. Resting behavior can occur while standing as well as lying down in sternal or lateral recumbency and is age-dependent. Adult horses spend 80% of their resting time standing and only a relatively small proportion of their 24 h time budget recumbent, while foals under three months of age lie down for 70–80% of their resting time [18,37,38,52,73,74]. Young domestic horses are lying down approximately 25% of the day [43,46], which is significantly more than adults that are recumbent for approximately 5% of the day (Table 3) [40,42]. Domesticated horses, analogous to wild horses, lie down mostly between midnight and four in the morning [39,46].

Measuring lying behavior is an essential component of equine welfare assessment because horses usually fall asleep shortly after lying down; thus, recumbency is a reliable indicator of sleep. Four stages of horses’ sleep-wake rhythm are typically differentiated and defined by specific cortical electronic activity and movement patterns: wakefulness, drowsiness, slow-wave-sleep, and paradoxical or rapid eye movement (REM) sleep, with the majority of sleep occurring between midnight and 5:00 am [39,44,46,60,72,75,76]. While sleep in horses is not uniquely associated with recumbency as horses can sleep standing, recumbency (sternal or lateral) is required for rapid eye movement (REM) sleep, which is vital for many physiological and cognitive functions [53,72]. Indeed, in other species including humans, REM-sleep deprivation has been linked to hyperalgesia and persistent chronic pain [53,77,78].

In horses, decreased lying time budgets were associated with inappropriate environmental conditions, stress, and painful musculoskeletal issues [4,40,50,52,53]. In contrast, increased lying times were observed in animals with higher social rank [79], larger stall size [80], straw bedding compared to shavings [81], and following administration of analgesics to horses suffering from orthopedic pain [4,52,53].

### 4.4. Time Budget for Locomotion and Movement

The time budget dedicated to movement or activity ranges from 4.3% to 13.4% in feral horses, with walk as the predominant gait and less movement at night than during the day (Table 3) [37,38,39]. In stallions, a greater need for rapid movement (trot, canter) has been reported [37]. The time budget for movement also includes, in addition to locomotion in the different gaits, play behavior which is most commonly observed in foals, and stereotypical movements such as weaving, crib-biting, and stall-walking. Horses with insufficient foraging opportunities and horses living in too densely stocked conditions show increased active locomotion patterns (Table 3) [4,26,27,42,45], confirming the value of locomotor activity as reliable indicators for horse welfare [82]. As horses also move while foraging, albeit slowly and less linearly, a clear distinction between foraging and movement behavior is required, but unfortunately this was not evident in all studies; thus, the time budgets for movement have to be interpreted with caution [9,15].

## 5. Conclusions

Activity time budgets allow an objective, quantitative on-farm welfare assessment and comparison of different management, feeding, and housing systems. In addition, changes in time budgets can be used to identify painful conditions and monitor the success of management interventions to improve equine welfare. However, the diversity of the horse populations, the environment the horses were living in, and the measurement methods leads to a great heterogeneity of the studies and is reflected in the highly variable time budgets. Thus, further studies of larger horse groups that live in clearly defined housing and management conditions, using modern observational technologies, such as biotelemetry or AI-based video analysis, are needed to further validate and establish the use of time budgets as a reliable indicator of equine welfare.

## Figures and Tables

**Table 1:** List of the included articles, their study design, observation method, the observation method(s), and the numbers of observation days.

Author(s) and Publication Year	Study Design	Control Group	Intervention	Observation Method(s)	Observation: Number of Days
Boy & Duncan 1979 [18]	Observational study (field)	no	no	manual, scan sampling 5-min blocks	24 h for two day in two weeks each month
Duncan 1980 [37]	Observational study (field)	no	no	manual, scan sampling 5-min blocks	24 h for two day in two weeks each month
Duncan 1985 [38]	Observational study (field)	no	no	manual, scan sampling 5-min blocks	24 h two day in two weeks each month
Boyd et al. 1988 [39]	Observational study (field)	no	no	manual, focal sampling	24 h
Berger et al. 1999 [28]	Observational study (field)	no	no	telemetry system ETHOSYS	one year
Price et al. 2003 [40]	Prospective non-blinded, non-randomized controlled trial	yes	elective arthroscopy	CVI, time lapse video recorder, manual, instantaneous sampling	continuously 72 h
Elia et al. 2010 [41]	Prospective non-blinded, non-randomized case series	no	feeding test	video time-lapse mode, manual, instantaneous sampling, Observer TM program	24 h 3 days per week
Aristizabal et al. 2014 [42]	Prospective non-blinded, non-randomized case series	no	two feeding devices	video, manual	24 h
Sartori et al. 2017 [43]	Prospective non-blinded, non-randomized case series	no	two isoenergetic diets	video, manual, focal sampling continuous	2 days
Maisonpierre et al. 2019 [44]	Prospective non-blinded, non-randomized case series	no	no	accelerometer (Actigraph)	24 h, 20 days
Correa et al. 2020 [45]	Observational study (stable)	no	additional hay bags	video, manual, ad libitum sampling, pedometer	24 h
Raspa et al. 2020 [46]	prospective non-blinded, non-randomized case series	no	three stocking density’s	video, manual, scan sampling at 15 min intervals	3 consecutive days (72 h)

**Table 2:** Signalment of horses included in the study. Depending on the data available in the respective papers, ages are provided as range, median (plus range), or mean ± standard deviation. Similarly, the gender is detailed depending on the information provided in the papers.

Author(s) and Publication Year	Horses (*n*)	Feral/Semiferal/ Domesticated	Breed	Gender	Age
Boy & Duncan 1979 [18]	11	semiferal, free-ranging	Camargue	Foals	n.a.
Duncan 1980 [37]	18	semiferal, free-ranging	Camargue	4 males, 9 mares, 5 yearlings	n.a.
Duncan 1985 [38]	18	semiferal, free-ranging	Camargue	9 males, 9 mares	n.a.
Boyd et al., 1988 [39]	8	semiferal, semi-reserve	Przewalski	1 stallion, 6 mares, 1 foal	n.a.
Berger et al. 1999 [28]	4	semiferal, semi-reserve	Przewalski	mares	n.a.
Price et al. 2003 [40]	12	domesticated	mixed breed	6 geldings, 6 mares	9 (4–15) years
Elia et al. 2010 [41]	8	domesticated	mixed breed	8 mares	(6–14) years
Aristizabal et al. 2014 [42]	10	domesticated	mixed breed	4 geldings, 6 mares	23 (20–27) years
Sartori et al. 2017 [43]	20	domesticated	Italian draft horse	10 males, 10 mares	12–18 months
Maisonpierre et al. 2019 [44]	6	domesticated	mixed breed	4 geldings, 2 mares	14 (4–22) years
Correa et al. 2020 [45]	7	domesticated	Brazilian jumper	3 males, 4 mares	10 ± 5 years
Raspa et al. 2020 [46]	22	domesticated	Comtois	19 males, 3 mares	22 ± 2 months

**Table 3:** Time budgets for standing/resting, lying, eating, movement/locomotion and other behaviors determined in the 12 studies included in this review, provided as range, or mean ± standard deviation.

Author(s) and Publication Year	TB in % Standing or Resting	TB in % Lying	TB in % Eating	TB in % Movement/Locomotion	TB in % Other Behaviour e.g. Drinking, Playing	Comments
Boy & Duncan 1979 [18]	8.1–11.8%	2.7–15%	13–62%			Data of foals during developing
Duncan 1980 [37]	12.9–19.52%	4.25–13.76%	50.82–63.89%	5.45–9.3%	n.a.	TB ranges based on the TB detailed in Table 9 of the publication, TB variation due to sex and age
Duncan 1985 [38]	13.4–29.3%	4.2–15.5%	60.8–66.6%	4.3–13.4%	n.a.	TB ranges based on the TB detailed in Table 8 of the publication; TB depending on season and gender
Boyd et al. 1988 [39]	36.6% ± 5.4%	5.3% ± 2.5%	46.4% ± 5.9%	7.4% ± 1%	10.2% ± 0.5%	Variation during daytime and season for feeding and standing
Berger et al. 1999 [28]	36.4% ± 15.7% winter: 48.4% ± 15% summer: 30.7% ± 29%	n.a.	29.8% ± 13%	n.a.	n.a.	Fluctuation over 24 h for feeding behaviour
Price et al. 2003 [40]	54 ± 9: control group 66 ± 12: post-surgery	8 ± 6: control group 4 ± 6: post-surgery	34 ± 6: control group 20 ± 9: post-surgery	0.015 ± 0.005: control group 3 ± 2: post-surgery	2% ± 0%: control 2% ± 1%: post-surgery	Values 0–24 h post-surgery, horses are housed in a stable
Elia et al. 2010 [41]	in stall: pellet-fed group: 58% in stall: hay-fed group: 36.6% paddock: pellet-fed group: 47.5 % paddock: hay-fed group: 32.4%	n.a.	In stall: pellet-fed group: 10% In stall: hay-fed group: 64%	paddock: pellet-fed group:12.3% paddock: hay-fed group 19.1%	searching: in stall: pellet-fed group: 11.5% in stall: hay-fed group: 1.2%	TB are provided separately for the time spent in the stable resp. the paddock for both groups
Aristizabal et al. 2014 [42]	ground feeding: 68% ± 8.6%. feeder: 65% ± 8.2%	ground feeding: 3% ± 5.5% feeder: 5% ± 6.68%	ground feeding: 28% ± 5.5% feeder: 31% ± 8.4%	n.a.	n.a.	Increased hay intake during daytime
Sartori et al. 2017 [43]	15.58% ± 5.02%	25.72% ± 4%	32.47% ± 3.75%	15.32% ± 2.37%	11.31 ± 3.32%	Details for gender and diet
Maisonpierre et al. 2019 [44]	33% (27.5–31.1) daytime51% (47.1–55.2) night-time 36% (33.3–39.2) standard paddock 42.9% (36.6–47.1) small paddock	n.a.	60.8% (58.2 65–4) daytime 46.8% (43.3–50.2) night-time 50.8% (47.9–55) standard paddock 48.6% (42.9–56.7) small paddock	4.6% (3.7–6.9) daytime2.4% (0.8–3.4) night-time 4.1% (3.1–5.8) standard paddock 2.5% (1.9–4.2) small paddock	n.a.	TBs per paddock size were calculated based on the hours spent for each activity provided in the publication
Correa et al. 2020 [45]	Basal: 62.7% Hay bag: 65%	Basal: 10.7% Hay bag: 9.9%	Basal: 12.5%Hay bag: 15.9%		Abnormal behaviour: Basal: 9% Hay bag: 5.9%	Leisure combines movement, standing, investigation
Raspa et al. 2020 [46]	30.56% ± 6.56%	27.33% ± 2.05%	30.55% ± 3.59%	4.07% ± 1.06%	<2%	reduced stocking density increased locomotion and playing, this change in TB was not quantified

## Data Availability

Data is contained within the article.
